# Tuning iteration space slicing based tiled multi-core code implementing Nussinov’s RNA folding

**DOI:** 10.1186/s12859-018-2008-6

**Published:** 2018-01-15

**Authors:** Marek Palkowski, Wlodzimierz Bielecki

**Affiliations:** West Pomeranian University of Technology, Faculty of Computer Science, Zolnierska 49, Szczecin, 71-210 Poland

**Keywords:** RNA folding, Parametric loop tiling, Computational biology, Nussinov’s algorithm, Parallel computing, Tile size selection

## Abstract

**Background:**

RNA folding is an ongoing compute-intensive task of bioinformatics. Parallelization and improving code locality for this kind of algorithms is one of the most relevant areas in computational biology. Fortunately, RNA secondary structure approaches, such as Nussinov’s recurrence, involve mathematical operations over affine control loops whose iteration space can be represented by the polyhedral model. This allows us to apply powerful polyhedral compilation techniques based on the transitive closure of dependence graphs to generate parallel tiled code implementing Nussinov’s RNA folding. Such techniques are within the iteration space slicing framework – the transitive dependences are applied to the statement instances of interest to produce valid tiles. The main problem at generating parallel tiled code is defining a proper tile size and tile dimension which impact parallelism degree and code locality.

**Results:**

To choose the best tile size and tile dimension, we first construct parallel parametric tiled code (parameters are variables defining tile size). With this purpose, we first generate two nonparametric tiled codes with different fixed tile sizes but with the same code structure and then derive a general affine model, which describes all integer factors available in expressions of those codes. Using this model and known integer factors present in the mentioned expressions (they define the left-hand side of the model), we find unknown integers in this model for each integer factor available in the same fixed tiled code position and replace in this code expressions, including integer factors, with those including parameters. Then we use this parallel parametric tiled code to implement the well-known tile size selection (TSS) technique, which allows us to discover in a given search space the best tile size and tile dimension maximizing target code performance.

**Conclusions:**

For a given search space, the presented approach allows us to choose the best tile size and tile dimension in parallel tiled code implementing Nussinov’s RNA folding. Experimental results, received on modern Intel multi-core processors, demonstrate that this code outperforms known closely related implementations when the length of RNA strands is bigger than 2500.

**Electronic supplementary material:**

The online version of this article (doi:10.1186/s12859-018-2008-6) contains supplementary material, which is available to authorized users.

## Background

RNA structure prediction, or folding, is an important ongoing problem that lies at the core of several search applications in computational biology. Algorithms to predict the structure of single RNA molecules find a structure of minimum free energy for a given RNA using dynamic programming. Nussinov’s folding algorithm [1] uses the number of base pairs as a proxy for free energy, preferring the structure with the most base pairs.

Nussinov’s algorithm is compute intensive due to a cubic time complexity. Fortunately, it involves mathematical operations over affine control loops whose iteration space can be represented by the polyhedral model [2]. Thanks to the simple pattern of dependences, loop tiling techniques can be used to accelerate Nussinov’s folding.

Let *S* be an *N*×*N* Nussinov matrix and *σ*(*i,j*) be a function which returns 1 if (*x*_*i*_,*x*_*j*_) match and *i*<*j*−1, or 0 otherwise, then the following recursion *S*(*i,j*) (the maximum number of base-pair matches of *x*_*i*_,...,*x*_*j*_) is defined over the region 1≤ *i*<*j*≤*N* as

$$S(i,j) = \max\limits_{1 \leq\ i < j \leq N} \left\{ \begin{array}{ll} S(i+1, j-1) + \sigma(i,j)\\ \max\limits_{i \leq k < j}(S(i, k) + S(k + 1, j)). \end{array}\right. $$

and *S*(*i,j*) is zero beyond that region.

Listing 1 represents the loop nest implementing Nussinov’s algorithm. It consists of triply nested affine loops with two statements accessing to two-dimensional array *S*.



Loop tiling or blocking is a crucial program transformation, which offers a number of benefits. It is used to improve code locality, expose parallelism, and allow for adjusting parallel code granularity or balance. All those factors impact parallel code performance [3].

In paper [4], we presented loop tiling based on the transitive closure of a dependence graph for Nussinov’s algorithm. It is within the iteration space slicing (ISS) framework [5]. The key step in calculating an iteration space slice is to calculate the transitive closure of the data dependence graph of the program; then transitive dependences are applied to the statement instances of interest to produce valid tiles. The idea of tiling, presented in paper [4], is to transform (correct) original rectangular fixed tiles so that all target tiles are valid under lexicographic order. We demonstrated higher speed-up of generated tiled code (for a properly chosen size of original tiles) than that of code produced with state-of the-art source-to-source optimizing compilers. But that paper does not answer what is the best size of original tiles allowing for generation of tiled code demonstrating the maximal speed-up. In general, the number of combinations of possible tile sizes can be very large. For each tile size, it is necessary to generate tiled code, compile and spawn it, and finally carry out code profiling. This can result in very high expenses not allowing for discovering the best tile size in practice.

The goal of this paper is to present an approach allowing us to determine the best tile size maximizing tiled code performance to be applied in practice. This approach is based on parametric tiling.

Parametric tiling is more general, it allows for defining tile size with parameters instead of constants [3]. With fixed size tiling, a separate program must be generated and compiled each time when tile size is changed. In general, this can be very expensive. Thereby, parametric tiling is more flexible and time and cost saving when we deal with code locality analysis and tuning code for target architectures. However, most state-of-the-art compilation tools do not provide parametric tiling, they are able to generate tiled code for only fixed tile size. Parametric tiling is generally known to be non-linear, breaking the mathematical closure properties of the polyhedral model.

To our best knowledge, well-known tiling techniques and optimizing compilers are based on linear or affine transformations [6–8], for example, the-state-of-the-art PluTo compiler [6] generates tiled code applying affine transformations derived. However, PluTo can only generate tiled code when tile size is fixed.

PrimeTile [9] is the first system to generate parametrically tiled code for affine imperfectly nested loops. It uses a level by level approach to generate tiled code, with a prolog, epilog, and a full-tiles loop nest corresponding to each nesting level of the original code. But loop tiling is generated seamlessly in the affine transformation framework.

DynTile [10] utilizes wavefront parallelism in the tiled iteration space corresponding to the convex hull of all the statement domains of the input untiled code. Tiles are scheduled dynamically, i.e., at run time.

PTile [11] is an approach to compile-time generation of code for wavefront parallel tiled execution.

Although DynTile, PTile, and PrimeTile present very effective tiling for stencils, using affine loop transformations, they do not allow us to tile dynamic programming kernels efficiently, in particular, they fail to tile the innermost loop in the code implementing Nussinov’s algorithm [2]. We show in this paper that tiling of that loop is crucial to achieve high performance. Furtermore, known techniques of mono-parametric tiling [3] (tile sizes are multiple of the same block parameter) do not guarantee notable locality improvements for Nussinov’s algorithm. To our best knowledge, there does not exist any parametric loop tiling scheme for the loop nest implementing Nussinov’s algorithm.

Mullapudi and Bondhugula presented dynamic tiling for Zuker’s optimal RNA secondary structure prediction [2] to overcome limitations of affine transformations. 3-D iterative tiling for dynamic scheduling is calculated by means of reduction chains. Operations along each chain find maximum and can be reordered to eliminate cycles. Their approach involves dynamic scheduling of tiles, rather than the generation of a static schedule.

Wonnacott et al. introduced serial 3-D tiling of “mostly-tileable” loop nests of Nussinov’s RNA secondary structure prediction in paper [12]. This approach tiles non-problematic iterations (iterations of loops ’*i*’ and ’*j*’) with classic tiling strategies while problematic iterations of loop (’*k*’) are peeled off and executed later. Unfortunately, the paper does not consider any parallel code, tiling is represented with serial code.

In this paper, we present an approach allowing for deriving the best size of original tiles to be used for generation of ISS based tiled code implementing Nussinov’s RNA folding.

## Methods

### Brief introduction

The *polyhedral model* is a mathematical formalism for analyzing, parallelizing, and transforming an important class of compute- and data-intensive programs, or program fragments consisting of (sequences of) arbitrarily nested loops. Loop bounds, statements conditions and array accesses are affine functions in the program.

Within the polyhedral model for analysis and transformation of affine programs, we deal with sets and relations whose constraints need to be affine, i.e., presented with linear expressions and constant terms. Affine constraints may be combined through the conjunction (and), disjunction (or), projection (exists), and negation (not) operators.

An access relation connects iterations of a statement to the array elements accessed by those iterations. Relations are defined in similar way as sets, except that the single space is replaced by a pair of spaces separated by the arrow sign →. We use the exact dependence analysis proposed by Pugh and Wonnacott [13], where loop dependences are represented with *relations*.

Standard operations on relations and sets are used, such as intersection (∩), union (∪), difference (-), domain (dom *R*), range (ran *R*), relation application (*S*^′^=*R*(*S*):*e*^′^∈*S*^′^ iff exists *e* s.t. *e* → *e*^′^ ∈ *R,e* ∈ *S*). The detailed description of these operations is presented in [13].

The *positive transitive closure* of a given lexicographically forward dependence relation *R*, *R*^+^, is defined as follows [5]:

$$\begin{array}{*{20}l} R^{+}=&\left\{e\rightarrow e':\ e\rightarrow e'\in R \ \vee\right. \\ &\qquad\qquad\left.\exists e^{\prime\prime}s.t.\ e\rightarrow e^{\prime\prime} \in R \ \land \ e^{\prime\prime}\rightarrow e'\in R^{+}\right\}. \end{array} $$

It describes which vertices *e* ^′^ in a dependence graph (represented by relation *R*) are connected directly or transitively with vertex *e*.

In sequential loop nests, the iteration *i* executes before *j* if *i* is *lexicographically less* than *j*, denoted as *i*≺*j*, i.e., *i*_1_<*j*_1_∨∃*k*≥1:*i*_*k*_<*j*_*k*_∧*i*_*t*_=*j*_*t*_,*for* *t*<*k*.

### Generation of tiles for the Nussinov loop nest

Let us recap tiled code generation for Nussinov’s algorithm presented in [4]. To generate valid 3-D tiled code for the Nussinov loop nest, we adopt the approach presented in paper [14], which is based on the transitive closure of dependence graphs.

The iteration domain of the Nussinov loop nest (see Listing 1) is represented with the following set.

$$Iteration\ Domain = \left\{ \begin{array}{ll} i : 0 \leq i \leq N - 1,\\ j : i+1 \leq j \leq N - 1, \\ k : \left\{ \begin{array}{ll} s1 : 0 \leq k \leq j-i-1, \\ s2 : k = 0.\end{array}\right. \end{array}\right. $$

Let vector ***I*** = (*i,j,k*)^*T*^ define indices of the Nussinov loop nest, diagonal matrix ***B*** = [ *b*_1_,*b*_2_,*b*_3_] define tile sizes, vectors ***II*** = (*ii,jj,kk*)^*T*^ and ***II*** ^′^ = (*iip,jjp,kkp*)^*T*^ specify tile identifiers. Each tile identifier is represented with a non-negative integer, i.e., the following constraint ***II*** ≥ 0 has to be satisfied.

First, we form parametric set, *TILE*(***II***, ***B***), including statement instances belonging to a parametric rectangular tile (parameters are tile identifiers) as follows

$${} TILE = \left\{ \begin{array}{ll} i : N-1-b_{1}* ii \geq i \geq max(-b_{1}*(ii+1),\\ \ \ \ N-1) \wedge ii \geq 0 \\ j : b_{2} * jj +i+1 \leq j \leq min(b_{2}*(jj+1) + 1,\\ \ \ \ N-1) \wedge jj \geq 0\\ k : \left\{ \begin{array}{ll} s1 : b_{3} * kk \leq k \leq min(b_{3}*(kk+1)-1, \\ \ \ \ \ \ j-i-1) \wedge kk \geq 0\\ s2 : k = 0. \end{array}\right. \end{array}\right. $$

*TILE_LT* (*TILE_GT*) is the union of all the tiles whose identifiers are lexicographically less (greater) than that of *TILE*(***II***, ***B***):

*TILE_LT* (*GT*) ={[***I***] |∃***II***^***′***^ s. t. ***II***^***′***^ ≺ (≻) ***II*** ∧***II*** ≥ 0 ∧B*II+***LB*** ≤***UB*** ∧***II***^***′***^ ≥ 0 and B*II^***′***^+***LB*** ≤***UB*** ∧***I*** in *TILE*(***II***^***′***^, ***B***)}.

For calculating exact relation *R*^+^, where *R* is the union of all dependence relations extracted for the Nussinov loop nest, we apply the algorithm presented in paper [15]. Next, we calculate the following set

$$TILE\_ITR = TILE - R^{+}(TILE\_GT), $$

which does not include any invalid dependence target, i.e., it does not include any dependence target whose source is within set *TILE_GT.*

The following set

$$\begin{array}{*{20}l} TVLD\_LT =& (R^{+}(TILE\_ITR) \cap TILE\_LT)\\&- R^{+}(TILE\_GT) \end{array} $$

includes all the iterations that i) belong to the tiles whose identifiers are lexicographically less than that of set *TILE_ITR*, ii) are the targets of the dependences whose sources are contained in set *TILE_ITR*, and iii) are not any target of a dependence whose source belong to set *TILE_GT*. Target tiles are defined by the following set

$$TILE\_VLD = TILE\_ITR \cup TVLD\_LT. $$

Next, we form set *TILE_VLD_EXT* by means of inserting into the first positions of the tuple of set *TILE_VLD* elements of vector ***II***: *ii*_1_,*ii*_2_,...,*ii*_*d*_. Nonparametric tiled code is generated by means of applying any code generator allowing for scanning elements of set *TILE_VLD_EXT* in lexicographic order, for example, isl AST [16].

In paper [4], we discuss parallelization of ISS based fixed tiled code by means of loop skewing which honors all dependences among generated tiles.

### Assumption about good original tile size and tile dimension

The most important step in generating target ISS based tiled code is defining an original tile size and dimension to form set *TILE* according to the approach presented in paper [4]. They impact serial and parallel code locality and performance. It worth noting that in general, target tiles represented with set *TILE_VLD* are different from original rectangular ones defined with set *TILE*. Target tiles can be parametric non-rectangular ones, i.e., the number of statement instances within such tiles depends on parametric upper loop index bounds.

For parametric tiles, it does not guarantee that the data size per a tile is smaller than the capacity of cache, this leads to decreasing code locality. The number of target parametric tiles and the percentage of the iteration space, occupied by them, depend on an original tile size. So, we strive to choose such original tile size which minimizes the percentage of the iteration sub-space occupied with target parametric tiles. Let us note that if for a given loop nest statement, the set (*R*^+^(*TILE_GT*) ∩ *TILE*) is empty, this means that for this statement, every target tile is the same as the corresponding original one, i.e., target parametric tiles are absent, so we have a good tiling scheme.

An additional file presents sets (*R*^+^(*TILE_GT*) ∩ *TILE*) for *s*1 when *B*=[7,79,133] and *B*=[1,79,133], respectively [see Additional file 1]. The set (*R*^+^(*TILE_GT*) ∩ *TILE*) for statement *s*2 is empty.

Scrutinizing the constraints of the set (*R*^+^(*TILE_GT*) ∩ *TILE*) for statement *s*1 when *B*=[7,79,133] allows us to conclude that most target tiles are different from original ones and they are non-rectangular. For many target tiles, the data size per a target tile can be greater than the cache capacity of a multi-core platform used by us for carrying out experiments (for details, see the next section). So, the 3-D tiling scheme for ISS based tiled code is not desired.

When we tile only the two inner loops, i.e., *B*=[1,79,133], we can derive the following conclusions. A value of parameter *b*3 has the most impact on the percentage of statement instances within non-corrected (rectangular) target tiles because it influences two loop indexes: *j* and *k*. For example, if the constraint *N*−*ii*+*b*2∗*jj*<=*j*<=78+*N*−*ii*+*b*2∗*jj* or *k*>*b*2∗*jj* is not satisfied, statement instances defined with vector (*i,j,k*)^*T*^, where *j,k* do not satisfy the above constraints, are all within rectangular tiles. Analyzing the constraints above, we may conclude that increasing the value of *b*2 increases the percentage of instances of statement *s*1 included in non-corrected target tiles. On the other hand, increasing this value leads to increasing data per a target tile and reducing parallelism degree. So, there exist “the golden mean” of *b*2, which maximizes target ISS based tiled code performance.

The value of *b*3 influences only one loop index, *k*, in the following constraints of the set (*R*^+^(*TILE_GT*) ∩ *TILE*): *k*>=*b*3∗*kk* and *k*<=*b*3−1+*b*3∗*kk*. Increasing the value of *b*3 increases the percentage of instances of statement *s*1 included in non-corrected target tiles. On the other hand, increasing this value leads to increasing the stride between cache lines which are referenced at each loop nest iteration (see Listing 1), this can dramatically reduce data reuse. So, a value of *b*3 cannot be large.

Summing up, we may expect that good original tiles are formed with the following matrix *B*=[1,*b*_2_,*b*_3_] and *b*_2_> *b*_3_, i.e., when we tile only the two inner loops. This assumption is confirmed by means of the results of our experimental study presented in the next section.



### ISS based parametric tiled code construction

To improve locality of tiled code, we use a model known as tile size selection (TSS) which can be classified into model-driven empirical search based. It is used to characterize and prune the space of good tile sizes. For each tile size in the pruned search space, a version of the program is generated and run on the target architecture, and the tile size with the least execution time is selected [17].

To apply TSS, we first form parametric 3-D tiled code to avoid generation and compilation of a separate code each time when tile size is changed.

For this purpose, applying our source-to-source optimizing compiler TRACO [18], we generate two nonparametric tiled codes for different values of elements of matrix ***B***=[*b*_1_,*b*_2_,*b*_3_] according to the technique presented in our paper [4]. We choose those values to be prime numbers to avoid generation of simplified nonparametric tiled code. We strive to generate tiled code whose structure is the same regardless of values of elements of matrix ***B***=[*b*_1_,*b*_2_,*b*_3_]. Next using those codes, we construct parametric tiled code.

An additional file presents generated tiled codes where *for* loops shown in violet correspond to ***B***_1_=[23,47,113], while *for* loops shown in red state for ***B***_2_=[37,79,167], [see Additional file 2]. Applying the way, presented in our paper [4], we prove that those codes are valid.

Analyzing those generated codes, we may conclude that i) their structures are the same, only integer factors, present in the same code position, are different; ii) there exist the following linear expressions defining the init-statement, condition, and iteration expression of *for* loops: *b*1+*b*2, *b*_2_,*b*_2_−1,*b*_2_+1,*b*_3_,*b*_3_−1; iii) there exists the non-linear expression of the form *b*1∗*b*2.

Taking into account the above conclusions, we form the following general linear model which is valid for each integer factor, say *y*, present in the expressions of tiled loop nest:

*y*=*a*_0_∗*b*_0_ + *a*_1_∗*b*_2_ + *a*_1_∗*b*_1_ + *a*_2_∗*b*_2_ + *a*_3_∗*b*_3_ + *a*_4_, where *a *_*i*_, *i*=0,..,4, are unknown integer coefficients, *b*_0_=*b*_1_∗*b*_2_.

Let us note that we replaced the non-liner expression *b*_1_∗*b*_2_ with the linear one *b*_0_.

We use the iscc calculator [19] to find unknown coefficients *a*_*i*_,*i*=0,1,2,3, in the above model as follows.

For each pair of values *y*_1_,*y*_2_, which appear in the same code position of the two generated nonparametric codes, we form a system of equations as follows

$${}\left\{ \begin{array}{ll} y_{1}=a_{0}*b_{01} \,+\, a_{1}*b_{21} + a_{1}*b_{11} + a_{2}*b_{21} + a_{3}*b_{31}\\ \qquad + \ a_{41},\\ y_{2}=a_{0}*b_{02} \,+\, a_{1}*b_{22} + a_{1}*b_{12} + a_{2}*b_{22} + a_{3}*b_{32}\\ \qquad+ \ a_{42}, \end{array}\right. $$

where *b*_*ij*_,*j*=1,2, are particular values of *b*_*i*_,*i*=1,2,3, for the first (*j*=1) and second (*j*=2) nonparametric codes, and apply the iscc calculator to resolve that system. It is worth noting that for each pair of *y*_1_,*y*_2_, the general model is simplified so that the resulting system includes only at most two unknowns, the reminding ones are absent.

For example, in the codes presented in [see Additional file 2] in line 6, we have in the same code position integers 93 and 153. We build the following set according to the iscc calculator syntax [19] {[*a*1,*a*2]: 23∗*a*1+47∗*a*2=93 ∧ 37∗*a*1+79∗*a*2=153}.

The constraints of this set are the two linear equations with the two unknowns (*a*_1_,*a*_2_), they obtained from the general model. The iscc calculator returns the following solution {[2,1]}, i.e., *a*1=2,*a*2=1, and *a*0=*a*3=*a*4=0. Hence, in the parametric code in line 6, we insert the expression 2∗*b*1+*b*2.

Table 1 presents all solutions for integers available in the examined nonparametric codes. Using those solutions, we form the parametric code presented in Listing 2. In [see Additional file 2], that code is presented with dark lines.

**Table 1:** Finding integer coefficients *a*_*i*_, *i*=0…4, of the model *y*=*a*_0_∗*b*_0_ + *a*_1_∗*b*_2_ + *a*_1_∗*b*_1_ + *a*_2_∗*b*_2_ + *a*_3_∗*b*_3_ + *a*_4_, where *b*_0_= *b*1* *b*2

y_1_	y_2_	*a*_0_	*a*_1_	*a*_2_	*a*_3_	*a*_4_	Formula	Lines,
*b*_1,2,3_=[23,47,113]	*b*_1,2,3_=[37,79,167]							[see Additional file 2]
70	116	0	1	1	0	0	b1+b2	6, 10, 56
93	153	0	2	1	0	0	2*b1+b2	6
1081	2923	1	0	0	0	0	b1*b2	6
22	36	0	1	0	0	-1	b1-1	10
23	37	0	1	0	0	0	b1	10, 15, 43, 46, 56, 62
21	35	0	0	1	0	-2	b1-2	10
113	167	0	0	0	1	0	b3	40, 49, 59
112	166	0	0	0	1	-1	b3-1	49, 59
46	78	0	0	1	0	-1	b2-1	56
47	79	0	0	1	0	0	b2	10, 15, 21, 30, 34,
								40, 43, 49, 52, 59

For so obtained parametric code, inter-tile dependences are described with non-affine expressions, so we cannot prove its validity applying the way presented in paper [4]. However, we seek for the best tile size using the previously mentioned TSS technique, which envisages running tiled code for particular fixed values of *b*_*i*_,*i*=1,2,3. So before running each fixed tiled code, we are able to check its validity applying the way presented in paper [4] because all inter-tile dependences for such a code are affine.

## Results and discussion

To carry out experiments, we have used a machine with an Intel Xeon processor E5-2699 v3 (2.3 Ghz in base and 3.6 Ghz in turbo, 18 cores/36 threads, 576 KB L1 Cache for code and data separately, 4.5 MB L2 Cache and 45MB L3 Cache) and 128 GB RAM. All programs were compiled by means of the Intel C++ Compiler (*icc* 15.0.2) with the -O3 flag of optimization. To implement multi-threaded parallel processing, the OpenMP programming interface [20] was chosen.

We experimented with randomly generated RNA strands 1 of length 2200 and 5000, the size of the average and longest human mRNA, respectively. We examined also longer strands (up to 10000) to illustrate benefits of tiling the innermost loop nest.

We considered 20 possible tile sizes along each dimension from the set {1, 2, 4, 6, 8, 12, 16, 24, 32, 40, 48, 64, 96, 128, 150, 200, 256, 300, 400, 512}. This leads to the search space including 20^3^ = 8000 possible tile sizes.

To carry out experiments, we wrote a script which automatically fulfills the following tasks: i) chooses one tile size from the search space (values of *b*_*i*_,*i*=1,2,3), ii) checks the validity of the tiled code with the chosen tile size according to the way presented in paper [4], iii) spawns the tiled code with the chosen tile size, iv) measures execution time, v) repeats steps i) - iv) for each tile size within the search space and collects all execution times. It worth noting that parametric code compilation runs only one time that greatly reduces search time.

Table 2 presents execution time of serial ISS based tiled code for some tile sizes. The execution time of the original (untiled) loop nest is 12.28 seconds. The results show that tiling of the two innermost loops allows for reaching minimal execution time of 3.276 seconds, this results in the maximal speed-up of 3.7. Under speed-up we mean the ratio of original program execution time to that of tiled one. Tiling the outermost loop allows us to reduce time execution to only 6.65 seconds. It is worth noting that only 15 tile sizes in the examined search space lead to greater execution time than that of the original program (see the last lines in Table 2).

**Table 2:** Execution time (in seconds) of serial ISS based tiled code for some tile sizes, *N* =2200

No.	b1	b2	b3	Time
1	1	128	16	3.2760
2	1	128	8	3.2910
3	1	150	8	3.3264
4	1	128	12	3.3506
5	1	96	16	3.3602
6	1	128	24	3.3913
7	1	150	12	3.4042
8	1	128	6	3.4247
9	1	96	8	3.4357
10	1	200	16	3.4645
...	...	...	...	...
467	2	150	16	6.6576
...	...	...	...	...
*Execution time of the original code:*				12.2802
7985	400	1	1	12.2872
7986	400	48	1	12.3162
...	...	...	...	...
8000	1	1	1	24.8607

**Fig. 1:**
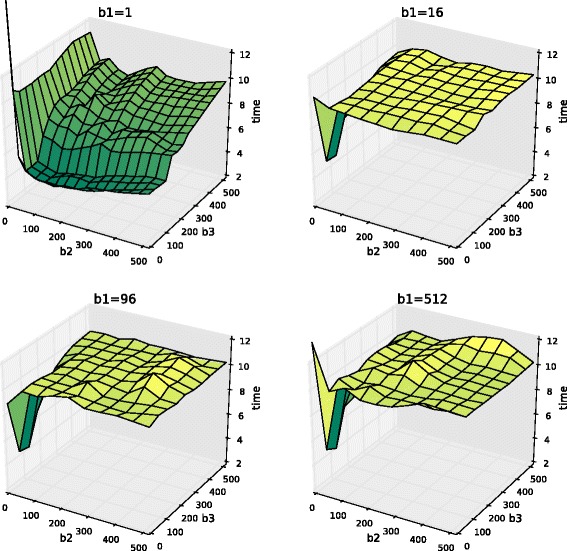
Execution time (in seconds) of serial ISS based tiled code, *N* = 2200, run on Intel Xeon E5-2699 v3. Results show that the maximal performance of serial ISS based tiled code is achieved when the outermost loop remains untiled (*b*_1_=1)

Figure 1 depicts execution times of serial ISS based tiled code for the four tile sizes of the outermost loop. As we can see, choosing *b*_1_=1 leads to the maximal tiled code performance. The explanation of this fact is presented in the previous sub-section. For this code, the best tile size within the examined search space is [1,128,16].

We carried out search of the best tile size in the same search space for multi-core tiled code with the bigger problem size, *N*=5000, and presented execution times in Table 3. For 32 threads, we observed super-linear tiled code speed-up of 112.9 for the tile size of [ 1×96×8]. The reason of super-liner speed-up is the cache affect resulting from the different memory hierarchies of the modern parallel computer used for carrying out experiments. Increasing the number of processors leads to increasing the size of accumulated caches from different processors. With the larger accumulated cache size, more or even all of the working data can fit into caches and memory access time reduces dramatically, which this considerably improve code locality.

**Table 3:** Execution time (in seconds) of parallel ISS based tiled code for some tile sizes, *N* = 5000, 32 threads used

No.	b1	b2	b3	Time
1	1	96	8	7.8751
2	1	150	12	8.0246
3	1	96	12	8.1903
4	1	128	12	8.1952
5	1	128	16	8.2199
6	1	128	6	8.2816
7	1	150	16	8.2831
8	1	50	8	8.3449
9	1	128	8	8.3841
10	1	96	6	8.4597
...	...	...	...	...
143	2	6	300	10.4351
...	...	...	...	...
*Execution time of the original code:*				334.3200
7993	2	1	2	335.3765
7994	1	2	2	411.5945
...	...	...	...	...
8000	1	2	1	889.6510

Obtained results show how much important is tiling of the innermost loop. To our best knowledge, such a tiling is not possible by means of optimizing compilers based on affine transformations. For example, the-state-of-the-art PluTo compiler (version 0.11.4) fails to tile the innermost loop of the examined program. The interesting fact is that the best code performance is achieved when the outermost loop nest remains untiled, tiling only the two innermost loops allows us to achieve better tiled code locality for the platform chosen for carrying out experiments. It is worth also noting that for the best tile size, the value of *b*_2_ has to be roughly tenfold bigger than that of *b*_3_. The explanations of those facts are given in the previous section.

**Fig. 2:**
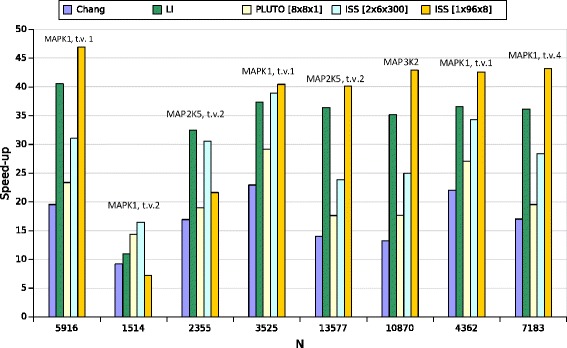
Speed-up of parallel codes for Nussinov’s matrix size of 5000 run on Intel Xeon E5-2699 v3. The horizontal coordinate represents the number of threads, the vertical one shows the speed-up of the examined codes

The results in Table 4, graphically presented in Fig. 2, demonstrate that our generated tiled code is scalable, i.e., increasing the number of threads increases code speed-up.

**Table 4:** Execution time (in seconds) of the Nussinov RNA folding codes for *N* = 5000 and different numbers of threads used

Threads	Original	Chang	Li	PluTo [ 8×8×1]	ISS [ 2×6×300]	ISS [ 1×96×8]
1	334.32	382.46	81.54	238.66	221.11	54.66
2		198.12	37.96	164.66	120.90	30.83
4		100.17	20.19	90.16	67.74	18.29
8		53.07	13.62	46.01	35.29	10.67
16		28.74	10.50	25.84	19.06	8.22
32		16.55	9.75	13.94	10.65	7.82

We compared the performance of ISS based tiled code with that of the manual parallel and cache efficient implementations [21, 22] of Nussinov’s RNA folding presented in Listing 3.

Chang et al. [21] modified Nussinov’s recurrences equations to simplify parallelization for multi-core architectures. RNA folding starts with initializing elements *S*(*i,i*) of the main diagonal of Nussinov’s matrix *S* and elements *S*(*i,i*+1) of the diagonal just above the main one, then elements of the remaining diagonals in the order *S*(*i,i*+2)…*S*(*i,i*+*N*−1) are calculated. All parallel threads synchronize before moving to the next diagonal.



Li et al. [22] suggested a cache efficient version of Chang’s code by using the lower triangle of matrix *S* to store the transpose of the computed values in the upper triangle of *S* [22]. They store *S*[*row*][*k*]+*S*[*k*+1][*col*] to variable *t* (line 19 instead of Chang’s line 16) and additionally store the value of *_max* to *S*[*row*][*col*] at the end of the loop body (line 25). Values of *S*[*k*+1][*col*] locate in the same column but values of *S*[*col*][*k*+1] locate in the same row, for *row*≤*k*<*col*. Li’s modifications accelerate rapidly code execution because reading values in a row is more cache efficient than reading values in a column [22].

Results in Table 4 show that our tiled code implementing Nussinov’s algorithm with the tile size [ 1×96×8] outperforms the implementations of Chang and Li (see Listing 3) for each examined number of threads (from 1 to 32) when *N*=5000.

This table includes also execution times of tiled code generated with the PluTo compiler, which tiles the two outermost loops^2^. The tile size [ 8×8×1] was chosen from many different tile sizes, examined by us, as one exposing the highest code performance. Those times are smaller than those achieved with Chang’s code. The cache efficient code proposed by Li et al. outperforms PluTo code and our 3-D tiled code. Only tiling of the two innermost loops allows us to achieve higher speed-up than that of Li’s implementation. Speed-up of the examined programs is depicted in Fig. 2.

**Fig. 3:**
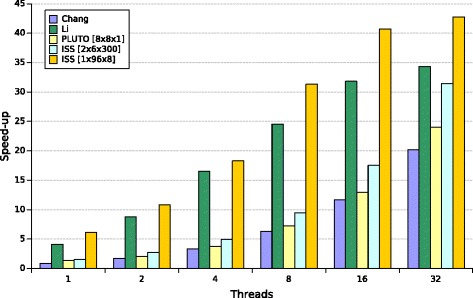
Speed-up of parallel codes run on Intel Xeon E5-2699 v3, 32 threads used. The horizontal coordinate represents Nussinov’s matrix size, the vertical one shows the speed-up of the studied codes. mRNAs acquired from the NCBI database

Furthermore, we studied code performance for different problem sizes defined as an RNA strand length, which is an important characteristic of Nussinov’s folding. We examined eight mRNAs of homo sapiens mitogen-activated protein kinase (MAPk) from the NCBI database^3^. Code execution times are presented in Table 5 while corresponding speed-up is depicted in Fig. 3. We observe that our code demonstrates higher speed-up than that of the reminding examined codes when the length of RNA strands is bigger than 2500. For short sequences (less than 2500) and 32 threads, related codes are faster (from 0,1 to 0,3 second per one strand) than ours. However, for short sequences, computation time is less than one second per one strand. The power of the presented approach is noticeable for longer strands, for example, our code for MAP2K6 variant 2 demonstrates 16 seconds time benefit per one strand against cache efficient Li’s code.

**Table 5:** Execution time (in seconds) of the Nussinov RNA folding codes for 32 threads and different lengths of RNA strands. mRNAs acquired from the NCBI database

mRNA definition	Lenght	Serial time	Chang	Li	PluTo [ 8×8×1]	ISS [ 2×6×300]	ISS [ 1×96×8]
MAPK1, trans. var. 1	5916	606,32	31,05	14,95	25,94	19,52	12,92
MAPK1, trans. var 2	1514	2,30	0,25	0,21	0,16	0,14	0,32
MAP2K5, trans. var 2	2355	15,57	0,92	0,48	0,82	0,51	0,72
MAP2K7, trans. var. 1	3525	102,32	4,46	2,74	3,51	2,63	2,53
MAP2K6, trans. var. 2	13577	6127,09	437,60	168,25	347,78	257,00	152,54
MAP3K2	10870	3033,73	229,53	86,30	171,54	121,44	70,68
MAP4K3, trans. var 1	4362	221,82	10,07	6,06	8,19	6,46	5,21
MAP4K4, trans. var. 4	7183	926,43	54,43	25,65	47,40	32,67	21,45

The performance improvement of the code generated with the presented technique against that of Li’s code for longer sequences is reached due to i) application of a tiling technique, which allows for increasing parallel code coarseness and locality, ii) choice of the optimal original tile size in the defined search space. All those factors together lead to significant improvement in code performance.

Summing up, we may conclude that the efficiency of cache reuse provided with ISS based tiled code becomes a dominant factor in achieving high code performance despite code complexity. Although our tiled code is more complex than the examined ones, choosing the best original tile size allows for achieving higher performance in comparison with the related examined codes on the multi-core machine used for experiments.

## Conclusion

In this paper, we presented an approach which allows us to choose in a given search space the best original tile size and tile dimension for generation of serial and parallel ISS based tiled codes implementing Nissinov’s RNA folding. Those codes are generated using the transitive closure of dependence graphs – the transitive dependences are applied to the statement instances of interest to produce valid tiles. Such a technique is within the well-known iteration space slicing framework.

Analyzing the constraints of a set representing valid target tiles, we make an assumption about good original tile size and tile dimension and confirm this assumption with carrying out experiments. The key step of this approach is constructing parallel parametric code, where variables defining tile size are parameters. The usage of parametric code allows us to compile target code only one time that significantly reduces search time.

The experimental study allows us to conclude that i) tiling the two innermost loops is the best tiling scheme for ISS based tiled code, i.e., the outermost loop has to be untiled; ii) the size of the second dimension of an original tile must be roughly tenfold bigger than the size of the third one.

Our implementation of Nussinov’s algorithm improves code locality and outperforms the serial original code by a factor of 3.7. We demonstrated super-linear speed-up of 112.9 for parallel code run with 32 threads. The tuned tiled code is more cache efficient than the closely related implementations of Li and Chang when the length of RNA strands is bigger than 2500 for the studied multi-core machine.

Under Nussinov’s algorithm conditions, the problem of folding a nucleotide sequence into a structure with minimal free energy becomes a simpler problem of finding a structure with the maximum number of base pairs [1]. Zuker et al. [23] refined Nussinov’s algorithm by using a thermodynamic energy minimization model, which produces more accurate results at the expense of greater computational complexity, but code implementing Zuker’s algorithm is affine. This allows us to apply the approach presented in this paper to that code. In future, we intend to apply our tiling strategies to generate parallel code implementing Zuker’s algorithm.

In future, we plan to engage heuristics and artificial intelligence methods in the tile size selection technique to reduce the search time of the best tile size. Furthermore, we plan to adopt the presented approach for multi- and many-core graphic cards using popular parallel processing interfaces.

## Additional files

Additional file 1 Set *R*^+^(*TILE_GT*) ∩ *TILE*. Presented in the ISL format. (PDF 4 kb)

Additional file 2 Construction of parametric code. Construction of parallel parametric 3-D-tiled code implementing Nussinov’s algorithm using two nonparameteric codes. (PDF 22 kb)

## Abbreviations

AST: Abstract syntax tree
ATF: Affine transformation framework
ISCC: Integer set counting calculator
ISS: Iteration space slicing
TSS: Tile size selection

## Footnotes

1 In paper [4], we demonstrate that tiled code performance does not change based on strings themselves, but it depends on the size of a string.

2 Wonnacot et al. widely demonstrated the weakness of tiling the two outermost loops for Nussinov’s algorithm in paper [12].

3
https://www.ncbi.nlm.nih.gov/
