# Supplementary material for: Tuning iteration space slicing based tiled multi-core code implementing Nussinov’s RNA folding
Source: BMC Bioinformatics. 2018 Jan 15;19:12. doi: 10.1186/s12859-018-2008-6 (PMC5769393; doi:10.1186/s12859-018-2008-6)
Supplement: Supplementary file 1 — Set R+(TILE_GT) ∩ TILE. Presented in the ISL format. (PDF 4 kb) [file 12859_2018_2008_MOESM1_ESM.pdf]

Set (R+(TILE_GT) intersection TILE) for s1
and B = [7, 79, 133]

[N, ii, jj, kk] -> { [i, j, k] : i >= -7 + N - 7ii and i >= 0 and j <= 79 + 79jj + iand j < N and k >= 0 and ((kk = 0 and k<= -4 - 79jj – i + j and k <= -137 - i + j and k <= -3 + N - 7ii - i) or (i <= -2 + N - 7ii and j >= 3 + 79jj + i and j >= 136 + 133kk + i and k >= 133kk and 0 < k <= 132+ 133kk) or (i <= -2 + N - 7ii and j >= 3 + 79jj + i and k >= 133kk and 0 < k <= -2 - i + j and k <= 132 + 133kk) or (i <= -2 + N - 7ii and j >= 2 + 79jj + i and j >= 135 + 133kk + i and 133kk <= k <= 132 + 133kk) or (i <= -2 + N - 7ii and j >= 2 + 79jj + i and k >= 133kk and 0 < k < -i + j and k <= 132 + 133kk) or (i < N - 7ii and k > 79jj and k >= 133kk and 0 < k < -i + j and k <= 132 + 133kk) or (i <= -3 + N - 7ii and j >= 4 + 79jj + i and j >= 4 + i and k >= 133kk and 2 <= k < -i + j and k <= 132 + 133kk) or (kk = 0 and k <= -2 - 79jj - i + j and k <= -135 - i + j and k <= -2 + N - 7ii - i) or (kk = 0 and k <= -2 - 79jj - i + j and k <= -2 - i + j and k <= -2 + N - 7ii - i)) }

Set (R+(TILE_GT) intersection TILE) for s1
and B = [1, 79, 133]

[N, ii, jj, kk] -> { [i = -1 + N - ii, j, k] : ii < N and kk >= 0 and N - ii + 79jj <= j <= 78 + N - ii + 79jj and j < N and k > 79jj and k >= 133kk and 0 < k <= -N + ii + j and k <= 132 + 133kk }

Set (R+(TILE_GT) intersection TILE) for s2 is empty
