# Supplementary material for: Tuning iteration space slicing based tiled multi-core code implementing Nussinov’s RNA folding
Source: BMC Bioinformatics. 2018 Jan 15;19:12. doi: 10.1186/s12859-018-2008-6 (PMC5769393; doi:10.1186/s12859-018-2008-6)
Supplement: Supplementary file 2 — Construction of parametric code. Construction of parallel parametric 3-D-tiled code implementing Nussinov’s algorithm using two nonparameteric codes. (PDF 22 kb) [file 12859_2018_2008_MOESM2_ESM.pdf]

Construction of parallel parametric 3-D tiled code implementing Nussinov's algorithm using the two nonparametric codes.

```
// 23, 47, 113      first nonparametric tiled code, violet lines
// 37, 79, 167      second nonparametric tiled code, red lines
// b1, b2, b3       target parameteric tiled code, black lines
for( c1 = 0; c1 <= floord(70 * N - 93, 1081); c1 += 1)
for( c1 = 0; c1 <= floord(116 * N - 153, 2923); c1 += 1)
for( c1 = 0; c1 <= floord((b1+b2) * N -(2*b1+b2), b1*b2); c1 += 1)
  #pragma omp parallel for                      // code without differences, green lines
  for( c3 = max(0, c1 - (N + 22) / 23 + 1); c3 <= min((N - 2) / 47, (23 * c1 + 21) / 70); c3 += 1)
  for( c3 = max(0, c1 - (N + 36) / 37 + 1); c3 <= min((N - 2) / 79, (37 * c1 + 35) / 116); c3 += 1)
  for( c3 = max(0, c1 - (N + b1-1) / b1 + 1); c3 <= min((N - 2)/b2, (b1 * c1 + b1-2) / (b1+b2)); c3+=1)
    for( c4 = 0; c4 <= 1; c4 += 1) {
      if (c4 == 1) {
        for( c7 = max(-N + 23 * c1 - 23 * c3 + 1, -N + 47 * c3 + 2); c7 <= min(0, -N + 23 * c1 - 23 * c3 + 23); c7 += 1)
        for( c7 = max(-N + 37 * c1 - 37 * c3 + 1, -N + 79 * c3 + 2); c7 <= min(0, -N + 37 * c1 - 37 * c3 + 37); c7 += 1)
        for( c7 = max(-N + b1 * c1 - b1 * c3 + 1, -N + b2 * c3 + 2); c7 <= min(0, -N + b1 * c1 - b1 * c3 + b1); c7 += 1)
          for( c9 = 47 * c3 - c7 + 1; c9 <= min(N - 1, 47 * c3 - c7 + 47); c9 += 1)
          for( c9 = 79 * c3 - c7 + 1; c9 <= min(N - 1, 79 * c3 - c7 + 79); c9 += 1)
          for( c9 = b2 * c3 - c7 + 1; c9 <= min(N - 1, b2 * c3 - c7 + b2); c9 += 1)
            for( c10 = max(0, 47 * c3 - c7 - c9 + 2); c10 <= 1; c10 += 1) {
            for( c10 = max(0, 79 * c3 - c7 - c9 + 2); c10 <= 1; c10 += 1) {
            for( c10 = max(0, b2 * c3 - c7 - c9 + 2); c10 <= 1; c10 += 1) {
              if (c10 == 1) {
                S[(-c7)][c9] = MAX(S[(-c7)][c9], S[(-c7)+1][c9-1] + sigma(-c7,c9);
              } else {
                if (N + 23 * c3 + c7 >= 23 * c1 + 2)
                if (N + 37 * c3 + c7 >= 37 * c1 + 2)
                if (N + b1 * c3 + c7 >= b1 * c1 + 2)
                  for( c11 = 0; c11 <= 47 * c3; c11 += 1)
                  for( c11 = 0; c11 <= 79 * c3; c11 += 1)
                  for( c11 = 0; c11 <= b2 * c3; c11 += 1)
                    S[(-c7)][c9] = MAX(S[(-c7)][c11+(-c7)] + S[c11+(-c7)+1][c9], S[(-c7)][c9]);
                for( c11 = 47 * c3 + 1; c11 < c7 + c9; c11 += 1)
                for( c11 = 79 * c3 + 1; c11 < c7 + c9; c11 += 1)
                for( c11 = b2 * c3 + 1; c11 < c7 + c9; c11 += 1)
                  S[(-c7)][c9] = MAX(S[(-c7)][c11+(-c7)] + S[c11+(-c7)+1][c9], S[(-c7)][c9]);
            } }
      } else
        for( c5 = 0; c5 <= 47 * c3  / 113; c5 += 1)
        for( c5 = 0; c5 <= 79 * c3 / 167; c5 += 1)
        for( c5 = 0; c5 <= b2 * c3/ b3; c5 += 1)
          for( c7 = max(-N + 23 * c1 - 23 * c3 + 1, -N + 47 * c3 + 2); c7 <= min(0, -N + 23 * c1 - 23 * c3 + 23); c7 += 1) {
          for( c7 = max(-N + 37 * c1 - 37 * c3 + 1, -N + 79 * c3 + 2); c7 <= min(0, -N + 37 * c1 - 37 * c3 + 37); c7 += 1) {
          for( c7 = max(-N + b1 * c1 - b1 * c3 + 1, -N + b2 * c3 + 2); c7 <= min(0, -N + b1 * c1 - b1 * c3 + b1); c7 += 1) {
            if (N + 23 * c3 + c7 >= 23 * c1 + 2) {
            if (N + 37 * c3 + c7 >= 37 * c1 + 2) {
            if (N + b1 * c3 + c7 >= b1 * c1 + 2) {
              for( c11 = 113 * c5; c11 <= min(47 * c3, 113 * c5 + 112); c11 += 1)
              for( c11 = 167 * c5; c11 <= min(79 * c3, 167 * c5 + 166); c11 += 1)
              for( c11 = b3 * c5; c11 <= min(b2 * c3,   b3 * c5 + b3-1); c11 += 1)
                S[(-c7)][(47*c3-c7+1)] = MAX(S[(-c7)][c11+(-c7)] + S[c11+(-c7)+1][(47*c3-c7+1)], S[(-c7)][(47*c3-c7+1)]);
                S[(-c7)][(79*c3-c7+1)] = MAX(S[(-c7)][c11+(-c7)] + S[c11+(-c7)+1][(79*c3-c7+1)], S[(-c7)][(79*c3-c7+1)]);
                S[(-c7)][(b2*c3-c7+1)] = MAX(S[(-c7)][c11+(-c7)] + S[c11+(-c7)+1][(b2*c3-c7+1)], S[(-c7)][(b2*c3-c7+1)]);
            } else
              for( c9 = N - 23 * c1 + 70 * c3; c9 <= min(N - 1, N - 23 * c1 + 70 * c3 + 46); c9 += 1)
              for( c9 = N - 37 * c1 + 116 * c3; c9 <= min(N - 1, N - 37 * c1 + 116 * c3 + 78); c9 += 1)
              for( c9 = N - b1 * c1 + (b1+b2) * c3; c9 <= min(N - 1, N - b1 * c1 + (b1+b2) * c3 + b2-1); c9 += 1)
                for( c11 = 113 * c5; c11 <= min(47 * c3, 113 * c5 + 112); c11 += 1)
                for( c11 = 167 * c5; c11 <= min(79 * c3, 167 * c5 + 166); c11 += 1)
                for( c11 = b3 * c5; c11 <= min(b2 * c3, b3 * c5 + b3-1); c11 += 1)
                  S[(N-23*c1+23*c3-1)][c9] = MAX(S[(N-23*c1+23*c3-1)][c11+(N-23*c1+23*c3-1)] + S[c11+(N-23*c1+23*c3-1)+1][c9], S[(N-23*c1+23*c3-1)][c9]);
                  S[(N-37*c1+37*c3-1)][c9] = MAX(S[(N-37*c1+37*c3-1)][c11+(N-37*c1+37*c3-1)] + S[c11+(N-37*c1+37*c3-1)+1][c9], S[(N-37*c1+37*c3-1)][c9]);
                  S[(N-b1*c1+b1*c3-1)][c9] = MAX(S[(N-b1*c1+b1*c3-1)][c11+(N-b1*c1+b1*c3-1)] + S[c11+(N-b1*c1+b1*c3-1)+1][c9], S[(N-b1*c1+b1*c3-1)][c9]);
    }     }
```
